# Graves’ hyperthyroidism in pregnancy: a clinical review

**DOI:** 10.1186/s40842-018-0054-7

**Published:** 2018-03-01

**Authors:** Caroline T. Nguyen, Elizabeth B. Sasso, Lorayne Barton, Jorge H. Mestman

**Affiliations:** 1 Division of Endocrinology, Diabetes, & Metabolism, Department of Medicine, Keck School of Medicine, University of Southern California, 1540 Alcazar Street, CHP 204, Los Angeles, Ca 90033 USA; 2 Division of Maternal Fetal Medicine, Department of Obstetrics and Gynecology, Keck School of Medicine, University of Southern California, 2020 Zonal Avenue, IRD 220, Los Angeles, CA 90033 USA; 3 Division of Neonatology, Department of Pediatrics, LAC+USC Medical Center, Keck School of Medicine, University of Southern California, Los Angeles, Ca 90033 USA; 4 Division of Endocrinology, Diabetes & Metabolism, Department of Medicine and Obstetrics and Gynecology, Keck School of Medicine, University of Southern California, 1540 Alcazar Street CHP 204, Los Angeles, California 90033 USA

**Keywords:** Hyperthyroidism, Pregnancy, Antithyroid drugs, Methimazole, Propylthiouracil, TRAb, Neonatal hyperthyroidism, Thyroid storm

## Abstract

**Background:**

Graves’ hyperthyroidism affects 0.2% of pregnant women. Establishing the correct diagnosis and effectively managing Graves’ hyperthyroidism in pregnancy remains a challenge for physicians.

**Main:**

The goal of this paper is to review the diagnosis and management of Graves’ hyperthyroidism in pregnancy. The paper will discuss preconception counseling, etiologies of hyperthyroidism, thyroid function testing, pregnancy-related complications, maternal management, including thyroid storm, anti-thyroid drugs and the complications for mother and fetus, fetal and neonatal thyroid function, neonatal management, and maternal post-partum management.

**Conclusion:**

Establishing the diagnosis of Graves’ hyperthyroidism early, maintaining euthyroidism, and achieving a serum total T4 in the upper limit of normal throughout pregnancy is key to reducing the risk of maternal, fetal, and newborn complications. The key to a successful pregnancy begins with preconception counseling.

## Background

Graves’ Hyperthyroidism (GH) is an autoimmune condition caused by antibodies stimulating the thyroid stimulating hormone receptor (TSHR). GH affects 0.2% of pregnant women [1]. Establishing the correct diagnosis and effectively managing GH in pregnancy is challenging; pregnancy alters thyroid physiology and laboratory testing, antithyroid drugs (ATDs) are associated with teratogenicity, and maternal, fetal, and newborn complications are directly related to control of GH and in a few cases to the levels of serum maternal thyroid-stimulating immunoglobulin (TSI) [2]. Fetal and neonatal hyperthyroidism occurs in 1% to 5% of women with active or a past history of GH and is associated with increased fetal/neonatal morbidity and mortality if not diagnosed and treated [3]. All women of reproductive age with GH or past history of GH should receive preconception counseling.

### Preconception counseling

Counseling should take into consideration the woman’s desired timeline to conception and include a discussion of the risks and benefits of all treatment options (medical therapy, 131-I radioactive iodine ablation (RAIA), and surgery). Women with GH should be advised to postpone conception and use contraception until GH is controlled. Women with difficult to control GH on high doses of ATD should consider definitive therapy (RAIA or surgery) prior to conception.

In women considering RAIA, a pregnancy test should be done beforehand and conception should be delayed for 6 months until the woman is euthyroid on levothyroxine replacement therapy. TSH Receptor Antibodies (TRAb), measurable in 95% of patients with GH, may increase and stay elevated for months to years after RAIA therapy [4, 5]. Persistently elevated TRAb levels during pregnancy are prognostic of fetal thyroid dysfunction [6]. Consequently, women with very elevated TRAb levels prior to conception may be better candidates for thyroidectomy. After surgery, TRAb levels decrease and normalize within months to one year [4, 5].

In women who continue with ATD treatment, methimazole (MMI) is generally preferred to propylthiouracil (PTU) because MMI is dosed once daily and PTU has an association with hepatotoxicity. However, PTU is recommended for the first trimester of pregnancy because its teratogenic effects are considered less severe than those of MMI. Switching from MMI to PTU in anticipation of conception should be considered. Alternatively, women may switch to PTU once pregnant.

Patients taking ATDs should use contraception and be counseled to pay close attention to menstrual cycles. A pregnancy test should be done immediately if there is a missed period. The risk of birth defects from ATDs is greatest during weeks 6–10 [7].

### Etiologies of hyperthyroidism

While the etiologies of hyperthyroidism are extensive (Table 1), GH and gestational transient thyrotoxicosis (GTT) account for the majority of hyperthyroidism in pregnancy. Distinguishing between GTT and GH is important because GTT is a transient, mild hyperthyroidism that does not require treatment with ATD and is not associated with adverse pregnancy outcomes [8].

**Table 1:** Causes of Hyperthyroidism in Pregnancy [33]

Thyroid Disease	
Graves’ disease	
Chronic thyroiditis	
Painless thyroiditis	
Subacute thyroiditis	
Toxic adenoma	
Multinodular goiter	
Non-autoimmune hyperthyroidism	
Gestational transient thyrotoxicosis	
Multiple gestations	
Trophoblastic disease	
Hyperplacentosis	
Hyperreactio luteinalis	
TSH receptor mutation	
TSH-producing pituitary adenoma	
Iatrogenic	
Excessive levothyroxine (LT4) intake	
Overtreatment	
Factitious	
Drugs	
Iodine	
Amiodarone	
Lithium	

#### Gestational transient thyrotoxicosis (GTT)

GTT affects 1–5% of pregnant women early in pregnancy and is not due to intrinsic thyroid disease [9]. As in GH, patients with GTT may present with palpitations, anxiety, tremors, and heat intolerance. A severe form of GTT is hyperemesis gravidarum, which is characterized by significant nausea, vomiting, and weight loss of up to 5 kg. These symptoms along with negative TRAb and the absence of Graves ophthalmopathy, goiter, and prior history of GH favor the diagnosis of GTT. The clinical course is closely correlated with hCG levels, which begin to rise at week 7 of gestation. Symptoms spontaneously resolve as serum concentration of hCG decreases between weeks 14 and 20 [10].

Women with twin pregnancies, another frequent cause of GTT, have higher levels of human chorionic gonadotropin (hCG) for a more prolonged period of time and consequently may be more symptomatic [11]. Trophoblastic disease (i.e., hydatidiform moles and choriocarcinoma) are also associated with elevated hCG levels and hyperthyroidism. Biochemically, GTT is characterized by a suppressed thyroid-stimulating hormone (TSH) and mildly elevated free thyroxine (FT4). TRAb are typically absent. TSH levels may continue to be suppressed for weeks after resolution of GTT. In very symptomatic patients a trial of beta-blocker may provide relief. In pregnancy, propranolol is preferred as atenolol has been associated with decreased birth weight [12, 13]. Women with a prior history of GTT have a higher likelihood of having a repeat episode [9].

#### Graves’ hyperthyroidism (GH)

As described above, the signs and symptoms of GH in pregnancy are similar to those of a non-pregnant GH patient. The diagnosis of GH should be suspected in a hyperthyroid pregnant woman who 1) was having symptoms prior to pregnancy 2) had a prior diagnosis of hyperthyroidism, and 3) had a previous birth to an infant with thyroid dysfunction.

The small increase in GH incidence and worsening of GH symptoms described in early pregnancy may be from the stimulation of the thyroid gland by hCG or elevation in TRAb levels during the first trimester [14–16]. As pregnancy progresses, changes in the immunologic response lead to improvement in symptoms. Postpartum, a rebound of the immune system can lead to exacerbation of GH [17].

### Thyroid function tests

In response to the rising hCG in early pregnancy, TSH concentrations may be below the non-pregnant reference range in up to 10% of normal pregnant women with 0.5–1% of women with completely suppressed TSH levels [18].

In the latter half of pregnancy, increased thyroid-binding globulin (TBG) and decreased serum albumin concentration can affect the widely available FT4 automated immunoassays resulting in significant variability between assays [19–21]. FT4 concentration also continuously declines throughout pregnancy [21]. Consequently, assay and trimester-specific pregnancy reference ranges are necessary if FT4 is used.

During pregnancy, the total thyroxine (TT4) assay is more consistent between assays. An estrogen-driven increase in TBG leads to the steady rise of TT4 from the first trimester until mid-gestation when the TT4 plateaus. After week 16, the non-pregnancy reference range may be adjusted by a factor of 1.5 and used to assess thyroid status (i.e., 4.5–12.5μg/dL becomes 6.75–18.75μg/dL) [21].

In addition, the FT4 index (FT4I) is a reliable marker of free thyroxine status during pregnancy, correcting TT4 for alterations in TBG [21, 22].

Free and total triiodothyronine (FT3/TT3) are rarely useful in the diagnosis and management of GH in pregnancy. One exception is in the diagnosis of an autonomous functioning nodule predominantly secreting T3.

#### TSH receptor antibodies (TRAb)

TRAb is a general term to define an antibody that binds the TSHR. TRAb can stimulate, block, or be neutral to the TSHR. GH is due to antibodies that stimulate the TSHR. TRAb is useful in confirming the diagnosis of GH [5].

Preconception, the presence of elevated TRAb is prognostic of risk of relapse of GH or failing ATD cessation [23]. During pregnancy, TRAb crosses the placenta and may induce fetal hyperthyroidism. TRAb levels over 3 times the upper limit of normal are associated with hyperthyroidism in the fetus and newborn [5].

TRAb assays can be divided into two categories: 1) TSH-binding inhibiting Ig (TBI)/thyrotropin binding inhibitory immunoglobulin (TBII) are competition-based assays that detect all TRAb (stimulating, blocking, and neutral) in patients’ sera by their ability to compete for binding of the TSHR and 2) Thyroid-stimulating Ig (TSI) is a bioassay that detects cyclic adenosine monophosphate (cAMP) production in cells incubated with patients’ sera and measures stimulating TRAb [24]. More recently, bioassays now have the ability to differentiate between stimulating and blocking antibodies [25].

TBII has a 97% sensitivity and 99% specificity for GH [26]. While TBII is unable to differentiate between stimulating or blocking antibodies, in a patient who is thyrotoxic an elevated TBI is due to the presence of stimulating TRAb in the majority of cases.

While thyroid peroxidase antibody (TPO-Ab) positivity has been associated with increased risk for pregnancy loss and premature delivery in euthyroid women, it is a nonspecific marker for autoimmune thyroid disease with no prognostic value for the fetus and newborn in Graves’ Disease.

### Pregnancy-related complications

Poor control of thyrotoxicosis is associated with pregnancy loss, pregnancy-induced hypertension, prematurity, low birth weight, intrauterine growth restriction, stillbirth, thyroid storm, and maternal congestive heart failure [27–29]. Women with uncontrolled GH are 9.2 times more likely to have neonates with low birth weight compared to non-GH women. However, if disease is controlled during pregnancy, they are 2.3 times as likely. Mothers with uncontrolled GH are also 16.5 times more likely to undergo preterm delivery and 4.7 times more likely to develop severe preeclampsia compared with women whose GH was controlled [30]. The rate of fetal demise and stillbirth in mothers with poorly controlled GH is 5.6% [31]. Persistent maternal TBII levels > 5 IU/L (approximately 3× ULN) in the latter half of pregnancy predicted neonatal hyperthyroidism with 100% sensitivity and 43% specificity (Table 2) [32].

**Table 2:** Risk factors for complications associated with hyperthyroidism in pregnancy

Risk factors [27, 28, 30]
• Long-standing GH • Gestational hypertension • Twin pregnancies • Anemia • Frequent infections (i.e., UTIs) • Late presentation to obstetric clinic • Inconsistent use of medications • Abnormal outcomes in prior pregnancies
Possible complications
• Maternal [28] ○ Miscarriages ○ Gestational hypertension ○ Preeclampsia ○ Congestive heart failure ○ Thyroid storm • Obstetrical ○ Premature delivery [82] ○ Placental abruption ○ Premature rupture of membrane ○ Gestational Hypertension • Fetal ○ Congenital malformations [83] ○ Hyperthyroidism ○ Developmental dysplasia of the hip associated with first-trimester maternal hyperthyroidism [84] ○ Intrauterine growth restriction (IUGR) ○ Small for gestational age (SGA) infants ○ Prematurity ○ Stillbirth • Neonatal ○ Prematurity ○ Hyperthyroidism [32] ○ Neonatal central hypothyroidism [71] ○ Low birth weight [30]

### Maternal management

ATDs remain the treatment of choice for GH during pregnancy. The lowest dose of ATD needed to maintain TT4 1.5× the upper limit of the non-pregnant reference range or FT4I at the upper limit of the reference range should be used. We have observed that normalization of FT4I can lag behind FT4 during ATD therapy leading to unwarranted larger doses of ATDs [33]. Care must be taken to avoid overtreatment with ATD. TSH may remain suppressed during ATD therapy even when TT4 of FT4I has normalized. The ATD dose should be lowered if TSH becomes detectable [33, 34]. “Block and replace” therapy, which consists of using an ATD in combination with levothyroxine therapy, is not recommended [29].

In the first trimester, PTU can be dosed at 50–150 mg every 8 h depending on the severity of the patient’s symptomatology. When switching from MMI to PTU, a ratio of 1:20 is used (i.e., MMI 15 mg = 300 mg of PTU per day dosed as 100 mg PO every 8 h) [2]. After the first trimester, MMI 5-20 mg can be given as a single dose. Occasionally, in very symptomatic patients up to 30-40 mg can be used daily. Propranolol 10-20 mg every 6–8 h can be used to control hyperadrenergic symptoms and tapered and discontinued as tolerated. Long term treatment with ß-blockers has been associated with poor intrauterine growth, fetal bradycardia, and neonatal hypoglycemia [35]. TSH, FT4/FT4I, or TT4 should be checked every 2–4 weeks as clinically indicated and doses of ATDs titrated based on clinical and biochemical response. ATD may be discontinued in women with mild disease requiring low dose of ATD and mildly elevated TRAb. In 30–40% of women, ATD may be discontinued after 30–34 weeks of gestation [34]. Fetal hypothyroidism is an indication to decrease or stop ATD.

#### Measurement of TRAb in pregnancy

TRAb should be checked in a pregnant woman with a past history of GH or active GH [36, 37]. If TRAb levels are low or undetectable in early pregnancy, no further TRAb testing is recommended [2]. If maternal TRAb is elevated or patient is being treated with ATDs, TRAb should be measured again between weeks 18–22. In those with levels near 3-4× above upper limit of normal (ULN), TRAb should be checked again during weeks 28–34. Maternal TRAb serum concentration greater than 3 times the upper limit of the reference range in the third trimester is a risk factor for neonatal hyperthyroidism [32, 38–40].

#### The role of thyroidectomy

Thyroidectomy in the second trimester is an effective option if a woman is unable to tolerate ATDs, non-consistent with drug therapy, requiring very high doses of ATDs, has a large goiter, or allergic to ATD. ß-blockade should be used to prepare patients for surgery and continued after surgery. A few days of SSKI or saturated solution of potassium iodide (50-100 mg/day) to help decrease the vascularity of the thyroid gland and control hyperthyroidism can be considered and is safe for the fetus [41].

#### Thyroid storm (TS) in pregnancy

TS is a rare complication of uncontrolled GH. Given its rarity, the incidence of TS in pregnancy is not clear. Davis et al. described 1 case in 120,000 deliveries over the span of 11 years at a single institution [27]. TS may occur when a chronically hyperthyroid patient encounters an additional stress or precipitating event (i.e., infection, preeclampsia, labor, surgery, or pregnancy) that leads to decompensation. Patients with TS present with tachycardia, thermic dysregulation, and altered mental status. If not adequately treated, TS can lead to multi-organ failure including congestive heart failure. Biochemical testing reveals suppressed TSH and elevated T4. Patients are best served in the ICU as they may require intubation and IV medications. Treatment entails adequate ATD, beta-blockade, and supportive care (Table 3).

**Table 3:** Management of Thyroid Storm [34, 63]

ATD management (decreases the synthesis and release of T4 and T3)	• PTU 100-150 mg PO every 8 h (PO, NGT) or • MMI 20 mg PO every 12 h (PO, NGT) or • MMI 40 mg in 200 cm^3^ water (Per rectum)
Non-selective beta blockade (symptomatic relief) to target: B_1_ – Heart rate B_2_ – Vasodilation B_3_ – Basal metabolic rate and heat production	• Propranolol 1 mg IV bolus followed by 1 mg/h (target heart rate of 90–100 bpm if adequately hydrated)
T4 and T3 release	• SSKI (potassium iodide) 5 drops or Lugol’s solution 10 drops every 8 h, 1 h after MMI (PO, NGT)
Generation of T3	• Decadron 4 mg IVPB every 6 h • PTU at above doses decreases peripheral conversion of T4 to T3
Incorporation of T4 and T3 into the nucleus	• L-carnitine 1-2 g twice a day [85]^a^
Fever	• Aspirin may increase thyroid hormones and acetaminophen can interfere with steroids. • Should improve with other treatment modalities.
Supportive care	• Antibiotics as infection common precipitating event • IVF –TS patients are at a fluid deficit. Fluid balance should be net positive. • Recommend against active cooling as can lead to peripheral vasoconstriction and hinder release of heat • Avoid aggressive use of diuretics. Intravascular depletion can lead to cardiovascular collapse • Low threshold to intubate

*PO* per oral, *NGT* nasogastric tube, *PR* per rectum, *IVF* intravenous fluids

^a^No studies in pregnant patients

### Anti-thyroid drugs: Complications for mother and fetus

ATD remains the mainstay treatment of women with GH during pregnancy. 3–5% of these women experience side effects. An allergic reaction is the most common side effect [42]. More severe side effects such as agranulocytosis (0.15%) and liver failure (< 0.1%) are rare [43, 44].

PTU and MMI are equally effective, cross the placenta at comparable rates, and may produce fetal hypothyroidism with or without goiter. Rates of fetal defects have been shown to be similar with both drugs; 2–3% with PTU and 2–4% with MMI [45–47]. Notably, this is similar to the overall 3% rate of major structural or genetic birth defects in the United States [48]. ATD associated defects are most common and severe in those exposed during weeks 6–10 [7, 45, 47, 49, 50]. PTU has traditionally been preferred over MMI in the first trimester because the birth defects associated with PTU are considered less severe and surgically correctable (Table 4) [51, 52]. Recently, a meta-analysis showed an increased risk of neonatal congenital malformations associated with MMI, but not PTU when compared to no ATD exposure [53]. However, other studies have not shown any difference in rates of congenital malformations between those treated with MMI or PTU and the general population [54].

**Table 4:** Birth defect associated with ATD

MMI
• Aplasia cutis • Choanal atresia • Esophageal atresia • Omphalocele • Urinary tract malformations • Eye defects • Ventral septal defects • Dysmorphic facies • Athelia • Developmental delay
PTU
• Pre-auricular sinus/fistula and cysts • Urinary tract abnormalities in males

### Fetal and neonatal thyroid function

The fetal (f) thyroid gland develops between 5 and 6 weeks gestation [55] and starts secreting thyroxine at 10 weeks of gestation [56]. Fetal thyroid hormone (fTH) production is limited until 18–20 weeks of gestation when fTSH receptors begin to function [57, 58]. fTSH levels will begin to rise until 28 weeks of gestation. fT4 levels will continue to rise until the end of pregnancy [59]. Prior to the onset of fTH production, the fetus relies on TH from the mother via transplacental passage [60].

Immediately after birth there is a rise in TSH, T4, and T3. TSH surges and peaks in the first 24 h of life and remains elevated for up to 3 to 5 days. T4 and T3 serum concentrations increase up to 6-fold within the first few hours of life, peaking at 24 to 36 h after birth [57, 59].Thyroid function tests gradually decrease to normal levels by 3 to 4 days of age [61].

#### Monitoring the fetus

Fetal risks in mothers with GH include hyperthyroidism due to inappropriate transplacental passage of maternal TRAb and hypothyroidism due to excessive maternal administration of ATD. During routine OB visits, antepartum surveillance should include assessment of fetal heart rate, typically performed with handheld doppler monitor. Fetal tachycardia is concerning for a thyroid etiology. Other causes of tachycardia such as infection, medications (cocaine, terbutaline), obstetric conditions (placental abruption, fetal bleeding), and fetal tachyarrhythmias should be ruled out [62].

#### TRAb titers and the fetus

As discussed previously, maternal serum TRAb titers provide useful prognostic information. TRAbs can cross the placenta and act to stimulate or block fetal thyroid hormone production once the fetal thyroid gland becomes functional [1, 2]. As stated above, persistent maternal TRAb levels greater than 3 times the ULN in the latter half of pregnancy predicted neonatal hyperthyroidism with 100% sensitivity and 43% specificity [32].

#### Indications for fetal ultrasound (FUS)

Women with a prior fetus or neonate with a thyroid disorder, TRAb greater than 3 times the ULN, fetal tachycardia, and poorly controlled hyperthyroidism should have a FUS. Initial US is generally performed at 18–22 weeks and then every 4 weeks to assess for gestational age, fetal viability, amniotic fluid volume, fetal anatomy, and detection of malformations [2, 63].

#### US findings of hyperthyroidism

The earliest sonographic sign of fetal thyroid dysfunction is fetal goiter, which appears as a solid neck mass [56, 64]. HR > 160 bpm for over 10 min, intrauterine growth restriction, presence of fetal goiter, advanced bone age, or oligo/polyhydramnios can also be seen in fetal hyperthyroidism [65]. Less commonly, fetal thyrotoxicosis can lead to heart failure, fetal hydrops, and fetal demise [63].

Fetal goiter can cause fetal, obstetric, and neonatal complications including polyhydramnios secondary to reduced swallowing ability, cervical dystocia, and mechanical obstruction of the fetal airway respectively [58, 66, 67]. Cesarean delivery may be preferred due to high risk of labor dystocia from a deflexed head [68].

#### Fetal hypothyroidism secondary to ATD drugs

Fetuses of mothers with GH on ATD can develop hypothyroidism and/or goiter due to overtreatment with ATDs [34]. Signs of hypothyroidism on FUS include fetal goiter, growth restriction, and delayed bone age [63]. ATD dose reduction or discontinuation should restore normal fetal thyroid function and decrease the size of the fetal goiter [56]. Rarely, intra-amniotic levothyroxine injections in conjunction with reduction of ATD dose may be indicated as combination therapy may lead to faster resolution of fetal goiter and recovery from hypothyroidism [67].

In the rare circumstance where the diagnosis remains unclear, cordocentesis remains the method of choice for confirmation of fetal thyroid status [1, 2, 57, 64]. Known as fetal blood sampling, cordocentesis involves US guided placement of a needle into the fetal circulation usually through direct placement into a free loop of umbilical cord or at the cord insertion site. Alternatively, TH concentrations in the amniotic fluid can be measured [58, 65]. While less hazardous, amniotic fluid hormone levels have not been validated as a reliable measure of fetal thyroid function [56].

Injection of levothyroxine into the umbilical vein should be restricted to cases of confirmed fetal hypothyroidism and progressive polyhydramnios in spite of repeated intra-amniotic injections [56, 69]. Overall risk of fetal complications associated with cordocentesis and intra-amniotic injection is low at 0.5% to 1% when performed at experienced centers [2, 57, 66]. The change in size of fetal goiter and extent of polyhydramnios help determine the response to treatment [64]. Repeat cordocentesis may be necessary to monitor therapy [70].

#### Fetal thyrotoxicosis

Isolated fetal hyperthyroidism in a euthyroid mother is treated with MMI 10-20 mg daily or larger doses if necessary after the first trimester with adjustment of the dose every few days based on fetal tachycardia and goiter size [34]. The lowest dose necessary to normalize the fetal heart rate (110–160 beats per minute) should be used [1]. Fetal assessment should then be performed every 1–2 weeks or as necessary with evaluation of fetal heart tones with hand-held doppler and US to assess growth, size of fetal thyroid gland, and amniotic fluid index. Care must be taken to prevent inducing fetal hypothyroidism due to excessive administration of ATD.

### Neonatal management

The neonatology team or treating pediatrician should be alerted regarding the mother’s diagnosis of thyroid disease before delivery [63]. Maternal use of ATDs during gestation, presence of TRAbs, and degree of control should be communicated directly. Progressive or complex thyroid illness during pregnancy warrants consideration of consultation with a pediatric endocrinologist prior to delivery [2].

Newborns of hyperthyroid mothers should be evaluated soon after birth. Neonates are at risk of hypothyroidism secondary to maternal ATD therapy, central hypothyroidism from untreated mothers via suppression of fetal TSH production that impairs the hypothalamic-pituitary-thyroid maturation [1, 71] and hyperthyroidism after the effect of maternal ATDs dissipates [33]. All newborns should be screened for thyroid dysfunction within 2 to 4 days after birth as early postnatal treatment reduces the risk of intellectual impairment [2].

TRAb can remain in the infant’s circulation for up to four months after delivery leading to postnatal thyrotoxicosis in 1–5% of infants of mothers with GH [34]. The diagnosis may be delayed by 48–72 h due to the transplacental passage of ATD medication [59]. FT4 measurement at birth should be repeated between days 3 and 5 of life and continuously followed if elevated [40]. TRAb positive neonates without biochemical or clinical evidence of thyroid dysfunction should have weekly clinical and laboratory follow up until the TRAb test becomes negative [40].

Some hyperthyroid neonates are first diagnosed at birth. Signs of neonatal thyrotoxicosis include tachycardia, tachypnea, pulmonary arterial hypertension, systemic hypertension and heart failure. They may also be small for gestational age with accelerated bone maturation and craniosynostosis [34, 63]. The recommended treatment regimen for neonatal hyperthyroidism is MMZ 0.5–1 mg/day with addition of propranolol (2 mg/kg/d) if severe [2]. PTU is not recommended as a first line treatment due to the high frequency of hepatotoxicity, liver failure and death, particularly in children [72]. Follow up is necessary until the hyperthyroidism resolves which may take months.

In cases of non-transient hypothyroidism, the recommended starting dose of levothyroxine is 10–15 mcg/kg/day in term neonates. Therapy should be initiated within two weeks of life for optimal outcomes and the infant should be followed for the first three years of life [2]. Most children do well with treatment and higher initial thyroxine dosage combined with shorter time to normalization contribute to improved neurodevelopmental outcome [61].

### Maternal post-partum management

GH mothers taking ATD at time of delivery should continue ATD after delivery even if breastfeeding. Those in remission are at risk for developing recurrent GH 4 to 12 months after delivery [73]. Elevated TRAb titers would favor relapse of GH. TSH and FT4 should be checked routinely the first year starting at 6 weeks after delivery.

GH in the postpartum period needs to be distinguished from postpartum thyrotoxicosis (PPT), a de novo autoimmune thyroid condition that occurs in approximately 5% of pregnancies in the first year postpartum [74]. PPT classically begins with a transient hyperthyroid phase followed by transient hypothyroidism before a return to euthyroidism. PPT does not require treatment with ATD. High TRAb titers would support relapse of GH. If the diagnosis remains unclear, 123-I RAIA uptake can help distinguish between GH and PPT in a non-breast-feeding woman [75]. If a woman is breastfeeding and a 123-I RAIA study must be done, the mother should discard the breast milk for 3–4 days [2].

#### Breastfeeding

Breastfeeding has been shown to be safe in mothers taking ATDs in appropriate doses. MMI and PTU both appear in breast milk in very small concentrations [76–78]. Studies of infants exposed to ATDs in breast milk in doses sufficient to control maternal GH had normal thyroid function and normal intellectual development [79, 80]. MMI is generally preferred in breastfeeding mothers because of hepatotoxicity associated with PTU. Experts recommend using the lowest effective doses possible with maximal doses of MMI 20 mg daily and PTU 300 mg daily [42, 81]. Doses should be split into 2 to 3 doses and administered after the mother has breastfed.

## Conclusions

The goal in the care of women with GH is the delivery of a euthyroid healthy newborn to a healthy mother. Establishing the diagnosis of GH can be challenging for the physician. Physical exam, history, and occasionally TRAb levels, can help distinguish GH from GTT, the most common cause of hyperthyroidism in pregnancy. Maintaining euthyroidism throughout pregnancy is key to reducing the risk of maternal, fetal, and newborn complications. ATDs remain the cornerstone of treatment of GH in pregnancy, with the lowest doses needed to maintain TT4 at 1.5× the upper limit of the non-pregnant reference range or FT4I in the upper limit of the reference range. PTU is preferred in the first trimester. In women with mild GH controlled on low doses of ATD with mildly elevated TRAb levels, ATD may be stopped in the last 4–8 weeks of pregnancy. Limited course of β-adrenergic blockers may be used to control symptoms of GH. Surgery in the second trimester may be indicated for women unable to take ATD, uncontrolled on high doses of ATD, or with large goiters. Persistently elevated levels of TRAb > 3× upper limit of normal is prognostic of fetal thyroid dysfunction and calls for close attention of the fetus and newborn. In the postpartum period, women are at risk for recurrence of GH symptoms and thyroid function tests should be evaluated at 6 weeks.

A multidisciplinary approach involving collaboration between the endocrinologist, maternal-fetal specialist, obstetrician, neonatologist, pediatric endocrinologist, and anesthesiologist is essential to the successful management of these complex patients. The key to a successful pregnancy begins with preconception counseling.

## Abbreviations

ATDs: antithyroid drugs
cAMP: cyclic adenosine monophosphate
f: fetal
FT3: free triiodothyronine
FT4: free thyroxine
FT4I: FT4 index
FUS: fetal ultrasound
GH: Graves’ Hyperthyroidism
GTT: gestational transient thyrotoxicosis
hCG: human chorionic gonadotropin
IUGR: intrauterine growth restriction
IVF: intravenous fluids
LT4: excessive levothyroxine
MMI: methimazole
NGT: nasogastric tube
PO: per oral
PR: per rectum
PTU: propylthiouracil
RAIA: 131-I radioactive iodine ablation
SGA: small for gestational age
TBG: thyroxine-binding globulin
TBI: TSH-binding inhibiting immunoglobulin
TBII: thyrotropin binding inhibitory immunoglobulin
TPO-Ab: Thyroid peroxidase antibody
TRAb: TSH Receptor Antibodies
TS: Thyroid Storm
TSH: Thyroid-stimulating hormone
TSI: Thyroid-stimulating immunoglobulin
TT3: total triiodothyronine
TT4: total thyroxine
ULN: upper limit of normal
